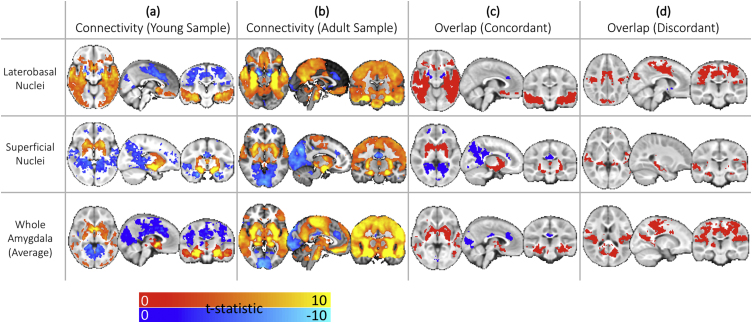# Human amygdala functional network development: A cross-sectional study from 3 months to 5 years of age

**DOI:** 10.1016/j.dcn.2018.06.004

**Published:** 2018-07-21

**Authors:** L.J. Gabard-Durnam, J. O’Muircheartaigh, H. Dirks, D.C. Dean, N. Tottenham, S. Deoni

**Affiliations:** a Division of Developmental Medicine, Boston Children’s Hospital, Harvard University, Boston, MA, 02115, USA; b Department of Forensic and Neurodevelopmental Sciences & Department of Neuroimaging, Institute of Psychiatry, Psychology and Neuroscience, King’s College London, London, UK; c Centre for the Developing Brain, Department of Perinatal Imaging and Health, School of Biomedical Engineering & Imaging Sciences, King’s College London, London, UK; d Advanced Baby Imaging Lab, Brown University School of Engineering, Providence, USA; e Waisman Center, University of Wisconsin-Madison, Madison, WI, 53702, USA; f Center for Healthy Minds, University of Wisconsin-Madison, Madison, WI, 53702, USA; g Department of Psychology, Columbia University, New York, NY, 10027, USA; h Department of Pediatrics, Warren Alpert Medical School, Brown University, Providence, USA

**Keywords:** Amygdala, Development, Early childhood, Resting-State, Functional connectivity, Infancy

## Abstract

Although the amygdala’s role in shaping social behavior is especially important during early post-natal development, very little is known of amygdala functional development before childhood. To address this gap, this study uses resting-state fMRI to examine early amygdalar functional network development in a cross-sectional sample of 80 children from 3-months to 5-years of age. Whole brain functional connectivity with the amygdala, and its laterobasal and superficial sub-regions, were largely similar to those seen in older children and adults. Functional distinctions between sub-region networks were already established. These patterns suggest many amygdala functional circuits are intact from infancy, especially those that are part of motor, visual, auditory and subcortical networks. Developmental changes in connectivity were observed between the laterobasal nucleus and bilateral ventral temporal and motor cortex as well as between the superficial nuclei and medial thalamus, occipital cortex and a different region of motor cortex. These results show amygdala-subcortical and sensory-cortex connectivity begins refinement prior to childhood, though connectivity changes with associative and frontal cortical areas, seen after early childhood, were not evident in this age range. These findings represent early steps in understanding amygdala network dynamics across infancy through early childhood, an important period of emotional and cognitive development.

## 1 Introduction

The amygdala plays a central role in the processing and production of emotional and social behaviour (Adolphs and Spezio, 2006; Ochsner et al., 2012; Phillips et al., 2003), both through its own activity as well as its dense anatomical and functional connectivity to the rest of the brain (Kim et al., 2011; Phelps and LeDoux, 2005; Young et al., 1994). Rich animal and human literatures examining the effect of both lesions and stress on the amygdala, across the lifespan, suggest that this region’s role in shaping emotion and social behavior is especially important during early post-natal development, with consequences for affective behavior lasting throughout life (Adolphs et al., 1994, 2002; Bauman et al., 2004; Bliss-Moreau et al., 2011, 2013; Graham et al., 2015a; Kazama et al., 2012; Prather et al., 2001; Raper et al., 2013; Shaw et al., 2004). Due to these associations, clarifying the role of the amygdala and its networks during early neurodevelopment is essential to understanding the ontogeny of affective phenotypes over typical and atypical development (Graham et al., 2015a; Roy et al., 2013; Shen et al., 2016; Tottenham and Gabard-Durnam, 2017).

For these reasons, much work in humans has focused on examining the developing amygdala and its networks. Structurally, the amygdala shows precocious development in utero. The structure of the amygdala is discernable in 8 week-old embryos, and cytoarchitectonic differentiation begins by 12 weeks post-conception (Nikolić and Kostović, 1986; O’Rahilly and Müller, 2006). The amygdala demonstrates mature structure by 8 months in utero (Ulfig et al., 2003). Postnatally, the amygdala undergoes rapid growth before 3 months of age (Holland et al., 2014; Uematsu et al., 2012), and prior studies report small or minor volume changes across childhood (Brenhouse and Andersen, 2011; Goddings et al., 2014; Mosconi et al., 2009; Nardelli et al., 2018; Østby et al., 2009; Uematsu et al., 2012). Similarly, the amygdala’s structural connections undergo early development. In utero, the amygdala demonstrates structural connectivity across the cortex, especially frontal and temporal regions, by 13 weeks post-conception (Vasung et al., 2010) Post-natal MRI in the first months has confirmed amygdala-frontal structural connectivity (Posner et al., 2016)and amygdala-subcortical and cortical structural connections are largely adult-like by early childhood, with some refinement of connections, especially subcortical and posterior connections, through adolescence (Saygin et al., 2015). Variance in amygdala volume and structural connection integrity have been related to cognitive and affective function in infancy through adolescence (Cismaru et al., 2016; Hanson et al., 2010; Mosconi et al., 2009; Ortiz-Mantilla et al., 2010; Swartz et al., 2014). This rich literature suggests the amygdala and its networks are already well developed structurally even shortly after birth.

The amygdala also demonstrates early functionality in humans. Studies have revealed a robust functional responsiveness to emotional stimuli by childhood (4–10 years) (Gee et al., 2013; Hare et al., 2008; Perlman and Pelphrey, 2011; Swartz et al., 2014; Thomas et al., 2001). In the absence of stimuli, the amygdala maintains ongoing functional connectivity with subcortical, limbic, and posterior regions in the neonatal and early infancy periods (Graham et al., 2016, 2018; Rogers et al., 2017). Moreover, variations in neonatal amygdala functional connectivity are associated with affective and cognitive profiles up to 2 years later (Graham et al., 2015a; Rogers et al., 2017). There is presently a gap in the literature with regard to amygdala function from the period of late infancy through early childhood, when substantial changes occur in affective and cognitive function (e.g. Brooks and Meltzoff, 2015; Feldman and Eidelman, 2009; Fox and Calkins, 2003; Tottenham and Gabard-Durnam, 2017). However, in childhood, the amygdala has been shown to exhibit functional connectivity with diffuse brain regions (including other subcortical and temporal/posterior cortical regions, Shen et al., 2016; Thijssen et al., 2017). Functional connections with other cortical regions, especially the prefrontal cortex, continue to develop from childhood into adulthood (Alarcón et al., 2015; Gabard-Durnam et al., 2014; Qin et al., 2012; Thijssen et al., 2017). Studies from infancy through adolescence suggest these amygdala reactivity and functional connectivity profiles are highly sensitive to environmental influences, both prenatally (Graham et al., 2018; Posner et al., 2016; Qiu et al., 2015) and postnatally (Achterberg et al., 2018; Gabard-Durnam et al., 2016; Gee et al., 2014; Thijssen et al., 2017). However, typical age-related changes in amygdala network development across infancy and early childhood are not yet robustly characterized.

An emerging focus in the study of the developing human amygdala is on understanding the early functional organization of the amygdala’s anatomical sub-regions. A large animal literature has established how these sub-regions serve different behavioral roles in maturity. Specifically, the laterobasal nuclei are central to aversive and appetitive valuation through cortical and subcortical connections (Ghashghaei and Barbas, 2002; Killcross et al., 1997; Pessoa and Adolphs, 2010). In contrast, the superficial nucleus processes olfactory and social information through connectivity with the insula and piriform cortex (Bzdok et al., 2013; Heimer and Van Hoesen, 2006; McDonald, 1998). Similarly, each of these sub-regions in humans has been shown to serve different affective functions through distinct activity and network connectivity (Ball et al., 2007; Davis et al., 1991; Etkin et al., 2009; Frühholz and Grandjean, 2013; Kerestes et al., 2017; Price, 2003; Roy et al., 2009; LeDoux, 2007). Preliminary developmental studies in older children (4–10 years) and adolescents suggest that progressive structural and functional segregation between these sub-regions’ cortical networks occurs throughout childhood and adolescence (Gabard-Durnam et al., 2014; Qin et al., 2012; Roy et al., 2013; Saygin et al., 2015). However, very little is known of the functional development of the human amygdala sub-regions across infancy and early childhood in typical development. This represents a significant gap in our understanding of this early-developing structure.

Therefore, the current study assessed amygdala functional networks in a cross-sectional sample from infancy through early childhood using resting-state functional magnetic resonance imaging (RS-fMRI). Specifically, we investigated early network connectivity of the whole amygdala as well as two of the amygdala’s major subdivisions, the laterobasal and superficial sub-regions, across infancy and early childhood. RS-fMRI provides a robust measure of functional network composition indexing the maintenance and stability of functional connections (Larson-Prior et al., 2009; Pizoli et al., 2011; Raichle, 2010). Additionally, RS-fMRI can facilitate the identification of developmental changes in functional networks as they emerge without task design confounds or confounds due to differences in cognitive functioning during the period of interest (Graham et al., 2015b; Uddin et al., 2010). Thus, RS-fMRI approaches enabled this study to begin addressing the paucity of understanding about human amygdala networks during early development.

## 2 Methods

### 2.1 Participants

The participants from this study were enrolled as part of a larger longitudinal study of typical brain development (Dean et al., 2014; Deoni et al., 2012a). Informed consent was obtained from each child’s parent or guardian in accordance to ethics approval from the Brown University Institutional Review Board. Participants were screened at enrollment to exclude major risk factors for developmental delay or psychopathology. Inclusion criteria included: uncomplicated single birth between 37 and 42 weeks gestation, no psychiatric or learning disorder diagnosis, no pre-existing neurological condition, no major head trauma, no abnormality detected on their fetal ultrasound, no reported in utero exposure to alcohol or illicit drugs (according to United States federal law, including cannabis, cocaine, opioids, amphetamine-type stimulants), and no first degree familial history of psychiatric or neurological illness as reported by caregivers. All data used in this analysis were acquired while the infant or child was sleeping naturally using previously reported imaging protocols and techniques (Dean et al., 2014). All scans took place at night, and all infants and children were asleep for 15–20 minutes before entering the MRI machine. A member of the study team remained present throughout the duration of the scan to monitor the participant.

**Fig. 1:**
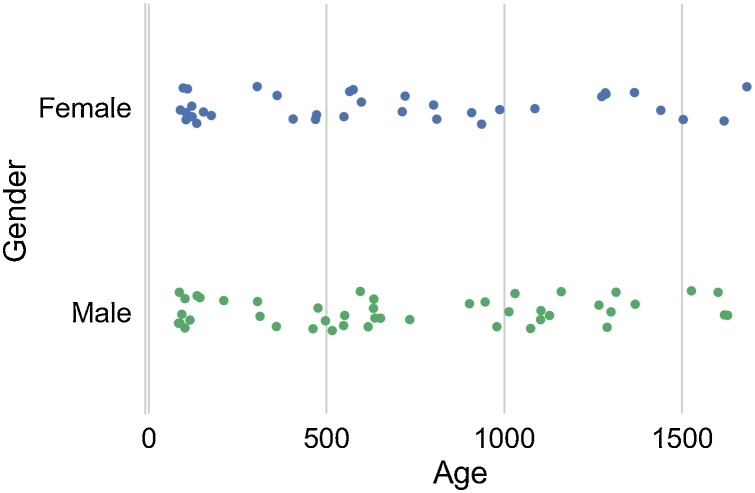
Age distribution of individual participants in this study, separated by sex. (For interpretation of the references to colour in this figure legend, the reader is referred to the web version of this article).

A total of 159 datasets were initially collected from 133 individual children between 3 and 54 months of age. Of the initial 159 datasets, 50 datasets were excluded based on an initial screening of motion characteristics of the functional data (see Functional MRI section below). A further 8 were excluded due to low quality of the structural scan. Finally, for children who were scanned on more than one occasion, only their later dataset was included, leading to the exclusion of a further 21 datasets. Unfortunately, the small number of longitudinal scans at present precluded robust mixed effects modeling of the data, so these additional scans were discarded for the present cross-sectional analyses (Ibrahim and Molenberghs, 2009). The remaining 80 participants were included in analyses. To account for variability in maturation at post-natal age due to varying gestational ages, all participants’ ages were corrected to a 40 week gestational period (i.e. corrected age), in keeping with recent studies of infant amygdala functional connectivity and pediatric research recommendations (Graham et al., 2016; Rogers et al., 2017). Therefore, infants with corrected ages between 84 and 1682 days contributed usable data for these analyses (see Fig. 1 for sample characteristics, stratified by participant sex). Of this sample of 80, self-reported ethnicity was collected for 70 subjects. Parents or guardians reported the ethnicity of their children as European American (40/70, 57.1%), African American (8/70, 11.4%), Asian American (1/70, 1.4%), mixed background (17/70, 24.3%) or other (4/70, 5.8%). Of these, 15/70 (21.4%) identified as Hispanic. Maternal and paternal education levels were scaled according to the Hollingshead scales of socioeconomic status, with 5 corresponding to at least 1 year of college education and 6 being a college or university graduate. Mean maternal education in the sample was a score of 5.86, and mean paternal education was a score of 5.4, indicating some college education for both parents (response range included scores between 4–7). Parental education was not included as a covariate in the neuroimaging models. In the subsample of participants with parental education data, neither maternal nor paternal education significantly correlated with age, the primary variable of interest in this study (p > 0.05, r < 0.04). This suggests, at least, that our age-related findings were not confounded by SES-related differences. Excluding parental education also prevented further sample size reduction in the imaging analyses.

### 2.2 Structural MRI

MRI data were acquired on a single Siemens 3 T Tim Trio equipped with a 12-channel radio-frequency head array. The T_1_ weighted SPoiled GRadient echo (SPGR) structural image used here was acquired as part of the mcDESPOT sequence (multi-component driven equilibrium single pulse observation of T1 and T2, (Deoni et al., 2008, 2012b)). This sequence is designed to enable calculation of the signal in water attributable to different tissue populations, but here we used only the high flip-angle T_1_ weighted image for registration and morphometry. The acquisition parameters for this sequence were optimized according to age group (Deoni et al., 2012c) and expected head size and images were acquired sagittally (www.cdc.gov/growthcharts/clinical_charts.htm). This also means that the acquisition parameters (for only this structural scan) varied with age (see Table 1). These changes in acquisition parameters provided a consistent (1.8 × 1.8 × 1.8) mm^3^ isotropic voxel volume resolution across all ages in the study. This approach mitigates confounds of age-varying spatial resolution in developmental studies and has been used in prior research focusing on age-related changes in brain connectivity (e.g. Dean et al., 2014; Deoni et al., 2014; O’Muircheartaigh et al., 2014).

**Table 1:** Acquisition parameters per age group in the paediatric sample.

Age Range	FoV (cm)	TE / TR (ms)	FA	Bandwidth (KHz per Voxel)	K-Space coverage
<9 Months	14*14*13	5.8 / 12	14	350	6/8 in the phase and slice encode directions
9–16 Months	17*17*14.4	5.9 / 12	14	350	6/8 in the phase and slice encode directions
16–28 Months	18*18*15	5.4 / 12	14	350	6/8 in the phase and slice encode directions
28–48 Months	20*20*15	5.2 / 11	16	350	6/8 in the phase and slice encode directions
>48 Months	20*20*16.5	4.8 / 10	18	350	5/8 in the phase and slice encode directions

Each T_1_-weighted image was first corrected for intensity non-uniformities using the N4BiasFieldCorrection tool (Tustison et al., 2010) and then stripped of non-brain tissue using the Brain Extraction Tool (Smith, 2002). The resulting image was non-linearly registered to a series of age-specific T_1_ weighted template images derived from an independent population of infants and toddlers who were scanned with the same sequence (Deoni et al., 2012a). This intermediate registration step to age-specific templates is common in studies of infant and toddler fMRI to improve registration quality and has been well-validated (e.g. Fonov et al., 2011; Gao et al., 2014; Graham et al., 2016; Qiu et al., 2015; Rogers et al., 2017; Sanchez et al., 2018). Images were then registered to an overall average pediatric brain template using the Advanced Normalization Tools (Avants et al., 2008). In this way, all images were normalized to the same pediatric space to facilitate group-level analyses.

### 2.3 Functional MRI

A gradient echo-planar imaging sequence was used for functional MRI data acquisition with the following parameters for all participants in the study (i.e. acquisition parameters did not change with age): repetition time = 2.4 s, echo time = 34 ms, flip angle = 80°, 32 interleaved 3.6 mm slices acquired at an orientation parallel to the anterior-posterior commissure line, in-place resolution of 2.97 mm, and a GRAPPA acceleration of 2. A total of 132 volumes were initially collected but the first 4 were discarded to allow magnetization to reach equilibrium leading to just over 5 min of data (307.2 s) per subject. Each subject contributed 307.2 s of data to analysis. That is, included scan length did not vary by age (due to the artifact correction procedures employed that do not censor entire volumes). Each resulting time-series was corrected for inter-scan motion using MCFLIRT (FMRIB Software Library (FSL), Oxford, United Kingdom (Smith et al., 2004) and had the middle functional scan rigidly registered to its T_1_ weighted image.

Following this pre-processing, motion characteristics were extensively examined. As this analysis focuses on an area near the edge of the brain and surrounded by vasculature and at risk of susceptibility signal dropout, the criteria used to exclude / include datasets were strict. First, scan-to-scan, absolute, and relative motion levels were assessed to discard datasets from further analysis if they contained too many contaminated volumes. For each time-series, the number of volumes with >0.2 mm scan-to-scan motion was recorded, consistent with prior infant studies of resting-state connectivity using 0.2 mm to 0.3 mm thresholds (Gao et al., 2014; Graham et al., 2015a; Rogers et al., 2017). Any time-series with >12 (i.e. 10%) of scans with these motion spikes (42 datasets) or with absolute or relative motion over the course of the time-series of >2 mm (8 datasets) were discarded from further analysis. The remaining datasets were corrected for structured noise associated with motion artifact using the AROMA package (Pruim et al., 2015) (v0.3b available at https://github.com/rhr-pruim/ICA-AROMA). AROMA is an independent component analysis (ICA) based method that automatically classifies and removes components identified as noise according to a combination the correlation of each components timeseries with motion, its spatial overlap with a mask at the edge of the brain, a mask of cerebrospinal fluid, and finally the proportion of high-frequency signal content. ICA-based motion correction approaches, including AROMA, are robust approaches for removing motion artifact in infant and child data (Ciric et al., 2017; Mongerson et al., 2017; Pruim et al., 2015; Rohr et al., 2018; Wylie et al., 2014). AROMA in particular has been shown to eliminate distance-dependent artifact without inducing false anti-correlations while preserving temporal degrees of freedom in pediatric data, pitfalls of another common motion correction approach including censoring and global signal regression (Ciric et al., 2017; Pruim et al., 2015). Given the extensive mature short- and long-range connectivity of the amygdala with patterns of both positive and anti-correlations (Roy et al., 2009) that we wanted to explore without bias in this developmental sample, the AROMA approach best facilitated these analyses. Noise components were detected and removed prior to any temporal filtering so the resulting cleaned data were temporally high-pass filtered using a 0.01 Hz filter. The resulting image was resampled into a 2 mm isotropic standard space and smoothed to a 5 mm isotropic full-width half maximum smoothness using 3dBlurToFWHM in Analysis of Functional NeuroImages (AFNI) software (Cox, 1996). This was to mitigate a recently discovered motion artifact of differential smoothness and ensure that all participants’ data had similar effective spatial smoothness (Scheinost et al., 2014).

Supplementary Fig. 2 shows the associations between participant age and common motion metrics (e.g. mean frame-to-frame motion) relevant to functional connectivity. These motion metrics were calculated on the data before they were corrected for motion through AROMA. There was a modest relationship between maximum absolute motion and age (r=-0.293, p < 0.05) but no significant relationship between age and mean relative scan-to-scan motion, number of motion spikes (scan-to-scan displacements larger than 0.2 mm), or maximum scan-to-scan motion. The motion-spike volumes were not explicitly censored as they were passed through AROMA, and the number of motion spikes was not related to any age-related changes in connectivity for any amygdala seed region (all p > 0.25). Inclusion or exclusion of the above motion covariates at the group level did not change average connectivity or their age-associations. Still, in keeping with recent recommendations for functional connectivity, at the level of between-participant models, average absolute scan-to-scan motion and average relative scan-to-scan motion for each participant were included as nuisance covariates to further address the insidious motion-related confounds faced in developmental functional connectivity studies.

**Fig. 2:**
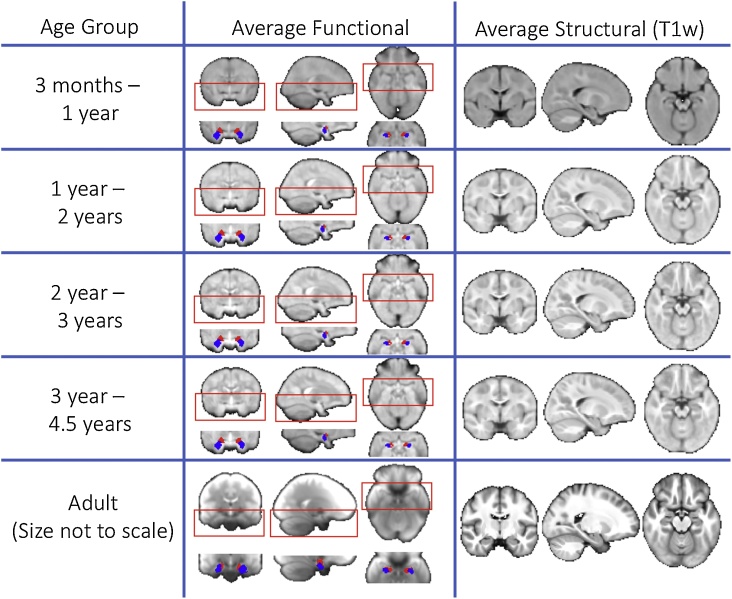
Average functional and structural (T_1_-weighted) images after spatial registration to a paediatric standard space. Images are shown at 4 different timepoints in the childhood dataset to illustrate registration performance and the placing of the amygdala seeds. Also shown is the average functional and structural image for the adult dataset in MNI space (note that the adult dataset was collected at a different site with different sequences). The bilateral superficial nuclei seeds are shown in red and the bilateral laterobasal seeds are shown in blue. (For interpretation of the references to colour in this figure legend, the reader is referred to the web version of this article).

### 2.4 Amygdala sub-regions of interest

Amygdala sub-regions, laterobasal and superficial, were derived from a population atlas of architectonic profiles transformed to a standard population MRI atlas (Amunts et al., 2005) provided with the FSL package. Importantly, the borders of amygdala subregions cannot be consistently mapped using macroanatomical landmarks from 3 T MRI, precluding the use of manual tracing approaches to identify amygdala subregions (Amunts et al., 2005; Solana et al., 2016). Instead, microscopic observation using post-mortem tissue is the gold standard method for delineating subregion boundaries, which was the approach taken to generate the subregion atlas used in the present study (Amunts et al., 2005). This atlas was thresholded and binarized at 50% probability for the adult analysis. For the paediatric functional analysis, this atlas was first transformed to this study’s paediatric template space and then thresholded at 50% probability. This threshold of 50% has been previously used for studies in both adults and young children (e.g. Alarcón et al., 2015; Ball et al., 2007; Gabard-Durnam et al., 2014; Qin et al., 2012; Roy et al., 2013). No sub-regions overlapped in space (see Fig. 2 for an image of the masks rendered on an average T_1_ weighted image and functional MRI image).

The amygdala’s precocious structural development in utero facilitates examination of infant amygdala subregions using the adult-derived atlas (Nikolić and Kostović, 1986; O’Rahilly and Müller, 2006; Ulfig et al., 2003). Cytoarchitectonic analyses have revealed that the amygdala is about 1500 mm^3^ unilaterally in the adult, larger than the partial volumes deduced from manual tracing with structural MRIs or extracted from standard atlases (e.g. Talairach, Harvard-Oxford atlases) (Amunts et al., 2005; Bzdok et al., 2013; Solano-Castiella et al., 2011). Studies performing manual amygdala segmentation with structural MRIs in neonates report volumes already about 750 mm^3^ unilaterally (e.g. Cismaru et al., 2016), and studies in early infancy that overlap in age with the present sample suggest volumes of 1000 mm^3^ or greater (Ortiz-Mantilla et al., 2010; Uematsu et al., 2012). That is, even the most conservative estimates of amygdala volume in infancy suggest the structure was of sufficient size in our youngest participants (3 months old) to measure with the thresholded cytoarchitectonic atlas. Adult atlases including the amygdala have been successfully registered to even younger infant data (i.e. from neonates) than the current sample as well (Gao et al., 2011). The amygdala LB and SF subregion volumes in the pediatric template space were sufficient sizes to reliably extract signal for functional connectivity analyses: the bilateral LB subregion was 2168 mm^3^ in pediatric space, and the smaller bilateral SF subregion was still 880 mm^3^. Accuracy of subregion registration was confirmed through visual inspection (see Fig. 2). Moreover, to ensure signal was extracted from the amygdala regions of interest with the same resolution across ages, spatial smoothness of the functional data was matched across all participants (i.e. spatial smoothness did not vary with age; Scheinost et al., 2014).

All sub-regions of interest were bilateral, given both the high degree of similarity between left and right amygdala functional connectivity maps previously reported in adults (Roy et al., 2009) as well as to facilitate comparison with prior studies in older developmental samples that used bilateral sub-regions of interest (Gabard-Durnam et al., 2014; Qin et al., 2012). A whole amygdala mask was calculated as the sum of the LB and SF masks. Supplemental analyses examining explicit differences between the right and left amygdala seeded connectivity did not return any significantly different (i.e. lateralized) connections in the pediatric sample (not reported). Prior work (e.g. Alarcón et al., 2015; Qin et al., 2012; Roy et al., 2009) has reported left and right amygdala seeded connectivity separately, but as there were no significantly different connectivity patterns between any left and right amygdala seeds in the current sample, bilateral amygdala results are reported. The use of bilateral seeds also reduced the number of overall models tested.

### 2.5 Functional connectivity

For the whole amygdala seed and for each amygdala sub-region individually, we calculated the mean time-series for every subject. Due to the proximity of the amygdala to areas of high vascular density, we also created a mask of regions associated with vascularization using a freely downloadable population atlas (Viviani et al., 2009) available here: http://www.uniklinik-ulm.de/struktur/kliniken/psychiatrie-und-psychotherapie/klinik-fuer-psychiatrie-und-psychotherapie-iii-ulm/home/forschung/clinical-neuroimaging/digital-atlas.html. See Supplementary Fig. 1 for the anatomical location of this mask overlaid on normalised EPI images.

We tested whole-brain functional connectivity with the amygdala in a single-subject general linear model (GLM). In a separate single-subject GLM, we also tested the two amygdala sub-regions together. The inclusion of both LB and SF subregion timeseries in a single GLM model facilitated the identification of average connectivity and age-related connectivity patterns unique to each subregion (i.e. unrelated to the other subregion’s connectivity). These models also included the timeseries of the vascular region as a confound regressor. For each subject, the GLMs were tested using FSL’s Feat (Smith et al., 2004).

A cross-sectional GLM was designed to test the average connectivity per contrast, including age, sex, average absolute scan-to-scan motion and average relative scan-to-scan motion as further covariates. Our contrasts of interest were average connectivity (whole amygdala, LB and SF) and relationships with age and sex. The resulting parameter estimates for the whole amygdala and the amygdala sub-region contrasts were then input to a higher-level group analysis using this GLM and tested by permutation using FSL’s Randomise. Multiple comparison correction for each contrast was performed using the cluster extent option in Randomise (thresholding based on the null distribution of cluster size), with an initial cluster forming threshold of t>3.1.

In addition, to explore non-linear age-related changes during this developmental period, two additional sets of GLMs were run as exploratory analyses, where the first set included a quadratic age term, and the second set included a natural log of age term. Both covariates were orthogonalised with respect to the linear age term. Results were corrected for multiple comparisons as before, using the cluster extent option in Randomise, with an initial cluster forming threshold of t>3.1. There were no statistically significant functional connectivity patterns associated with the quadratic age term that survived correction, so the following results are from set of analyses examining only the linear age-related and non-linear natural log(age) effects.

## 3 Results

### 3.1 Average functional connectivity with the amygdala

**Fig. 3:**
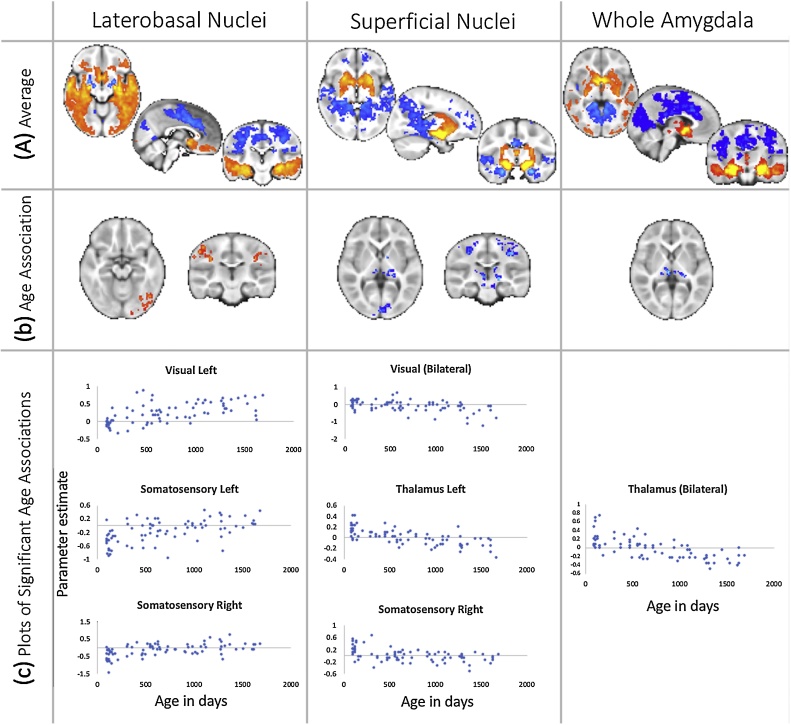
Average functional connectivity and linear age-related changes in connectivity with the amygdala. (A) Whole brain functional connectivity at rest between the subdivisions of the amygdala and the rest of the brain (two left columns) and the entire amygdala (rightmost column) in the early childhood sample. Images are shown after multiple comparison correction (p < 0.05, corrected for cluster extent). (B) linear associations with age between whole-brain functional connectivity and amygdala sub-regions and whole amygdala, middle row (hot colours denote positive association and cold negative). The plots (c) illustrate individual differences of connectivity strength (beta, y-axis) with age (in days, x-axis). (For interpretation of the references to colour in this figure legend, the reader is referred to the web version of this article).

The whole amygdala had average positive connectivity with ventral cortical and subcortical structures. Significant positive connectivity with cortical structures was limited to the temporal cortex (middle and inferior gyri), insula, and posterior orbitofrontal cortex (OFC), while connectivity with subcortical structures included the basal ganglia, striatum (ventral and dorsal), and thalamus (Fig. 3a). The whole amygdala had average negative connectivity with dorsal cortical regions. Significant negative connectivity was observed with visual cortex, precuneus, motor and somatosensory cortex, posterior, middle, and dorsal anterior cingulate cortex (Fig. 3a). For illustrative purposes, average amygdala (and sub-region) connectivity profiles in the younger and older halves of the sample are shown separately in Supplemental Fig. 3.

The LB sub-region had average positive connectivity with frontal and ventral cortical and subcortical regions in the paediatric sample (Fig. 3a). The cortical regions included bilateral OFC (medial and lateral), ventral medial PFC (vmPFC), superior frontal gyrus, motor and somatosensory cortices, temporal cortex (superior, middle, and inferior gyri), precuneus, and visual cortex (cuneus). Subcortical regions included extensive areas of the striatum (ventral striatum and putamen), the hippocampus and parahippocampal gyrus, the thalamus, and the cerebellum. The LB had average negative connectivity with distributed regions including bilateral dmPFC (supplemental motor area), dorsal ACC, pre- and post-central gyri, visual cortex (cuneus and lingual gyri), fusiform gyrus, and dorsal striatum (caudate).

The SF sub-region had predominantly average negative connectivity in the paediatric sample. However, positive connectivity was observed with subcortical structures including bilateral amygdala, thalamus, and striatum (ventral striatum, caudate, putamen) (Fig. 3a). The SF had average negative connectivity with bilateral rostral vmPFC (Brodmann Area 11), mPFC (Brodmann Areas 9,10), dorsal cingulate, supplemental motor area, middle frontal gyrus, precentral gyrus, superior and middle temporal gyri, supramarginal gyri, right-hemisphere angular gyrus, precuneus, cuneus, extensive posterior cingulate, fusiform gyrus, hippocampus and parahippocampal gyri, and thalamus.

No significant sex effects were found in any analyses of whole amygdala, LB, or SF functional connectivity during this developmental period.

### 3.2 Developing functional connectivity with the amygdala

**Fig. 4:**
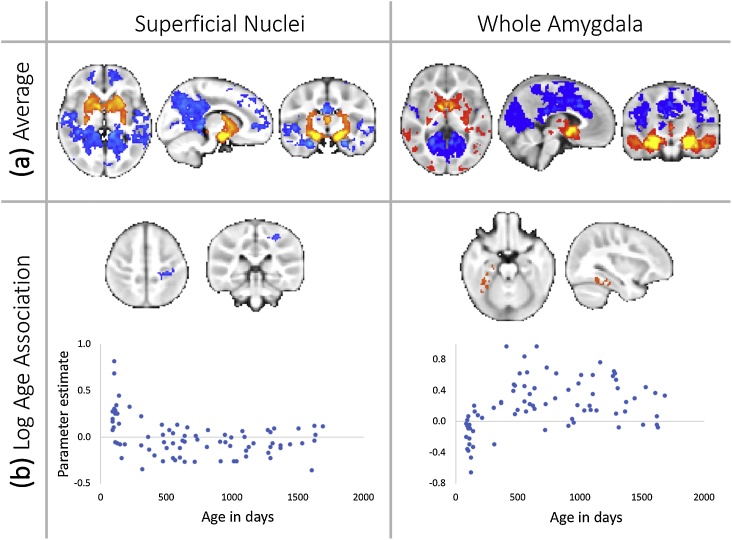
Non-linear age-related changes in amygdala functional connectivity. Average functional connectivity (a) and (b) non-linear age related changes in functional connectivity the superficial nuclei and the whole amygdala. Note that the relationship in motor cortex with activity in the superficial nuclei reduces to just below zero with age. No quadratic relationships were detected between age and functional connectivity. (For interpretation of the references to colour in this figure legend, the reader is referred to the web version of this article).

For the whole amygdala and amygdala sub-regions, associations between connectivity and increasing age were localized to sensory and motor-related regions (Fig. 3b and c). The whole amygdala showed decreasing connectivity with linear age for a region of bilateral thalamus, shifting from positive connectivity at age 3 months to negative connectivity by age 5 years (Fig. 3b and c). In addition, there was a small region of non-linear (natural log) age-related change in the right ventral temporal lobe, following the fusiform gyrus (Fig. 4b). When broken down to LB and SF connectivity, patterns of age-related change differed between structures.

The LB sub-region had positive linear age-related changes in connectivity across sensory and motor cortical regions, reflecting both reductions in negative connectivity and increases in positive connectivity. The LB sub-region showed a linear reduction in negative connectivity with the bilateral postcentral gyrus (somatosensory cortex), shifting from negative connectivity at age 3 months to no significant connectivity by age 5 years (Fig. 3b and c). The LB sub-region also showed increasing positive connectivity with linear age for a bilateral occipital cortex region, such that no statistically significant connectivity was observed at age 3 months, and positive connectivity emerged by age 5 years (Fig. 3b and c). There were no nonlinear (squared or natural log) age-related changes in LB connectivity observed during this developmental period.

In contrast, the SF sub-region showed negative age-related changes in connectivity across subcortical and cortical sensory and motor regions. The SF sub-region connectivity with the thalamus shifted linearly from positive connectivity at age 3 months to negative connectivity by age 5 years (Fig. 3b and c). Similarly, the SF sub-region connectivity with the somatosensory cortex shifted linearly from positive connectivity at age 3 months to negative connectivity by age 5 years (Fig. 3b and c). An overlapping SF-somatosensory cortical connection showed nonlinear (natural log) age-related decrease in connectivity during this period as well (Fig. 4b). The SF sub-region also showed linear, increasingly negative connectivity with occipital cortex, such that no statistically significant connectivity was observed at 3 months of age, and negative connectivity emerged by 5 years of age (Fig. 3b and c).

### 3.3 Supplementary analyses

Additional conjunction analyses directly compare the amygdala connectivity profiles in the pediatric sample to those of a publically available dataset of adult resting-state data (Supplemental Analyses, Supplemental Fig. 4). These analyses should be interpreted cautiously as the adult data were collected on a different machine from the pediatric sample with several different scan parameters, and were collected while the adults were awake (whereas the pediatric participants were all asleep). These analyses aim to facilitate preliminary comparisons between pediatric and adult connectivity patterns generated by similar data processing procedures until further research includes both infant and adult samples in the same study of amygdala functional connectivity (Supplemental Analyses, Supplemental Fig. 4).

### 3.4 Supplementary data

Average anatomical and functional images for four age groups in the pediatric sample (generated for illustrative purposes) are included (Supplemental Data). Average functional connectivity maps for the pediatric datasets as well as their slopes with age are also included as both raw t-statistic images, and the t-statistic images after correcting for multiple comparisons (masked by the significant clusters). All images are in nifti format.

## 4 Discussion

Despite the amygdala’s critical and basic role in shaping emotional and social behavior early in life, we currently have little knowledge regarding the human amygdala’s functional development during this period. Here, we characterized the functional (resting-state) networks of the amygdala and its LB and SF sub-regions. We also demonstrated their age-related changes in a large cohort of typically-developing children from 3-months to 5-years of age. We showed that the spatial pattern of functional connectivity pattern seen in later life is already largely present by early childhood (see supplemental analyses for comparison with adult connectivity), with the interesting exception of medial prefrontal cortex. Similarly, we distinguished functional connectivity patterns for each amygdala sub-region, suggesting network distinctions between sub-regions are evident and observable in early life. We have also shown age-related maturational changes in the whole amygdala and amygdala sub-regions’ resting-state connectivity limited to sensory and motor-related regions, a pattern unique to this early post-natal developmental period.

Distinct patterns of average connectivity and age-related changes in connectivity separated each of the amygdala sub-regions in infancy and early childhood, and were consistent with connectivity observed later in development. The observed pattern of both positive LB connectivity with temporal and ventral cortical regions and negative LB connectivity with dorsal and posterior cortical regions is highly consistent with stable patterns observed later in development and in maturity (Alarcón et al., 2015; Gabard-Durnam et al., 2014; Qin et al., 2012; Roy et al., 2013). Moreover, LB connectivity with sensory cortices that was discordant between the paediatric sample and a supplemental adult sample were largely the same connections showing age-related changes during this early developmental period in the direction of the mature adult phenotypes. A notable exception to this consistency was the observed LB-PFC connectivity. The LB has particularly robust anatomical connections with the vmPFC across species (Aggleton et al., 1980; Ghashghaei and Barbas, 2002; McDonald, 1998), but prior developmental studies have not consistently identified LB-vmpfc or LB-OFC functional connectivity (age-related increases across middle childhood and adolescence in Gabard-Durnam et al., 2014, but present in Qin et al., 2012 in middle childhood, and whole-amygdala OFC/vmPFC connectivity at preschool age reported by Shen et al., 2016). Here we show that LB-OFC and LB-vmPFC connectivity was already present in this sample of infants and young children, suggesting early, coordinated communication seems to occur between these regions. Functional connectivity between the LB and limbic regions, such as the vmPFC, striatum, and medial temporal lobe (e.g. hippocampus), has been implicated as a core network for stimulus valuation (Ghashghaei and Barbas, 2002; Pessoa and Adolphs, 2010), fear/threat memory, and conditioned response retrieval (LaBar and Cabeza, 2006; Maren and Quirk, 2004; Sierra-Mercado et al., 2011). Robust positive connectivity between the LB and all of these regions was already seen in this early developmental sample. Indeed, a recent report in 1-month olds has shown that amygdala-striatal and amygdala-vmPFC functional connectivity already correlate with later fear and cognitive development profiles (Graham et al., 2015a). The pattern of connectivity from our findings would suggest that connectivity with the LB sub-region (the largest cluster of nuclei), in particular, may be driving these amygdala results. LB-hippocampal-frontal functional connectivity also increases through adolescence, suggesting continued refinement of this circuitry occurs after this period of stable connectivity in infancy and early childhood (Gabard-Durnam et al., 2014; Qin et al., 2012; Silvers et al., 2017).

Age-related changes in LB connectivity were also observed during infancy and early childhood with sensori-motor and occipito-temporal regions. LB-occipital connectivity increased with age during infancy and early childhood, in parallel with reports of extensive changes in the face-processing capacities subserved in part by amygdala-occipito-temporal connections (Anderson et al., 2016; Cassia et al., 2009; Picozzi et al., 2009; Simion and Giorgio, 2015). Future work focusing on the emergence of this LB-occipito-temporal functional connectivity and face-processing capacity directly could elucidate whether this early connectivity drives these face-processing developments. The LB also showed refinement of connectivity with the sensorimotor cortex during infancy and early childhood. Specifically, negative connectivity between the LB and several regions within bilateral sensorimotor cortex in infancy was reduced to no significant connectivity between these regions. This selective reduction of LB connectivity with parts of the sensorimotor cortex may reflect spatial refinement of this functional connection during early development. Even so, the LB maintained significantly negative connectivity with other regions of sensorimotor cortex throughout this period. This finding is consistent with the negative connectivity maintained between the LB and regions of the sensorimotor cortex across childhood and adolescence into adulthood (Gabard-Durnam et al., 2014; Roy et al., 2009). Moreover, the LB-sensorimotor negative connectivity remaining after infancy has been observed to undergo strengthening (i.e. increasingly negative valence) during the childhood and adolescence periods (Gabard-Durnam et al., 2014). Together, the present and prior results suggest that LB-sensorimotor functional connectivity undergoes a series of refinements during a protracted developmental trajectory. Future studies including behavioral measures of motor development or tasks including motor responses during affective contexts may reveal the behavioral consequences of these different connectivity refinements across development.

SF patterns of functional connectivity and age-related changes in connectivity were also highly consistent with those identified in later development. In particular, the positive functional connections with ventral caudate and anterior putamen regions and negative connectivity with broad posterior-cingulate and superior-frontal regions that were concordant between the paediatric and adult samples have all been observed as stable patterns in older populations. The SF’s positive subcortical connectivity is also in line with preliminary studies in adult humans implicating the SF in olfactory and social incentive processing (Bzdok et al., 2013; Goossens et al., 2009; Heimer and Van Hoesen, 2006; Koelsch et al., 2013). Although positive SF connectivity extending to ventral cortical regions has been observed in older populations, this was not observed in the present sample, and it may be that this connectivity emerges in early childhood (at the junction of sample age-ranges between this study and prior studies) (Bzdok et al., 2013; Gabard-Durnam et al., 2014). It should be noted that the SF’s spatial proximity to ventricles tempers the extent of interpretation for this region’s connectivity at typical fMRI resolution.

The age-related changes in SF connectivity were also consistent with changes that continue through adolescence in older samples, suggesting contiguous, gradual refinement occurs in the SF-network. Specifically, while there was robust positive SF connectivity with much of the thalamus, consistent with both mature resting-state and stimulus-elicited SF-networks (Bzdok et al., 2013; Koelsch et al., 2013; Roy et al., 2009), there were also regions of the thalamus where connectivity with the SF changed to negative connectivity by early childhood, and thalamic connectivity continues to change (seen as positive connectivity attenuating) through adolescence (Gabard-Durnam et al., 2014). SF-occipital connectivity was also observed to change such that negative connectivity emerged by early childhood, mirroring the same shift from no SF connectivity to negative connectivity with other regions of the occipital lobe across childhood and adolescence (Gabard-Durnam et al., 2014; Roy et al., 2009). Lastly, SF-motor cortex connectivity in the left hemisphere changed from positive to negative connectivity by early childhood, foreshadowing a similar shift between SF and the right-hemisphere motor cortex that begins in middle childhood and persists into adulthood, although the mechanisms supporting this asymmetrical developmental timing remain to be explored. Although the interpretation of negative connectivity is unresolved, prior developmental studies of other functional networks have observed developmental shifts from positive to negative connectivity (e.g. Chai et al., 2014). Moreover, preliminary evidence from both empirical studies and simulations of functional network properties suggests that the emergence of negative connectivity reflects complex regulatory interactions between regions and networks, including reciprocal modulation between regions, suppression of one region by the other, and other inhibitory processes (Cabral et al., 2011; Gopinath et al., 2015; Parente and Colosimo, 2016, 2016). Future studies targeting SF function in specific stimuli contexts across infancy and early childhood can probe the nature of this potentially regulatory relationship with motor cortex.

Although functional networks continue segregating through adolescence, these spatial patterns of connectivity suggest core network components differentiating the sub-regions are in place early. This set of findings is consistent with evidence that amygdala sub-regions segregate structurally early in development (Humphrey, 1968; Payne et al., 2010; Ulfig et al., 2003; Saygin et al., 2015). Moreover, for areas where both amygdala sub-regions showed significant connectivity, frequently the two sub-regions demonstrated differently-valenced coupling (e.g. one sub-region showing positive connectivity, the other negative connectivity), suggesting the sub-regions have functional differentiation from each other for spatially-overlapping connections. For example, the putamen had robust connectivity with the LB and SF sub-regions, but this connectivity was negative with the LB and positive for the SF. In addition, sub-regions demonstrated distinct patterns of change in their network connectivity during this period. The LB switches to positive connectivity with sensori-motor regions, while the SF switched to negative connectivity by early childhood that continue through adolescence. These results suggest the sub-regions may have disparate trajectories of connectivity development in terms of both timing and network composition beginning in infancy.

Several patterns emerged across the sub-regions’ connectivity in infancy and early childhood distinguishing this period from amygdala networks in later developmental periods. First, the period of infancy is unique in that amygdala reactivity is absent to stimuli during this time that elicit responsiveness in childhood, but we and others have observed extensive functional connectivity during infancy (Blasi et al., 2011; Goksan et al., 2015; Graham et al., 2013, 2015a; Qiu et al., 2015; Rogers et al., 2017; Scheinost et al., 2016; Posner et al., 2016). Thus, future work may examine whether this early established connectivity facilitates the emergence of later amygdala reactivity by childhood. Secondly, no age-related changes were observed with any sub-region and association cortex during this early period, contrary to later childhood and adolescence (Tottenham and Gabard-Durnam, 2017; Qin et al., 2012). Together with previous studies, these results suggest that amygdala-subcortical and sensory-cortex connectivity begin refinement prior to childhood and continues through adolescence, while connectivity changes with associative and frontal cortical areas may begin after early childhood (Gabard-Durnam et al., 2014; Qin et al., 2012; Roy et al., 2013; Thijssen et al., 2017; Achterberg et al., 2018; Graham et al., 2018; Rogers et al., 2017; Scheinost et al., 2016).

In particular, positive connectivity of the whole amygdala or either sub-region was not observed during infancy and early childhood with the peri-genual medial anterior cingulate. This finding is consistent with other reports in infancy (Scheinost et al., 2016; Posner et al., 2016). These results stand in contrast to recent reports of amygdala resting-state connectivity in early childhood (Shen et al., 2016; Park et al., 2018), although differences in how the amygdala seeds were defined and the age ranges between the samples may in part account for these differences. Moreover, the present finding is consistent with other well-powered reports in middle childhood (Thijssen et al., 2017; Achterberg et al., 2018) and with prior cross-sectional and longitudinal reports that positive connectivity between the amygdala and this perigenual mPFC region underlying emotion regulation emerges in late childhood and adolescence (Alarcón et al., 2015; Gabard-Durnam et al., 2016; Qin et al., 2012). Aside from differences in data processing approaches across studies, given Thijssen and colleagues’ finding that amygdala-perigenual mPFC connectivity differs as a function of parent-child dynamics, and Achterberg and colleagues’ finding that amygdala-mPFC connectivity is largely shaped by environmental rather than genetic factors, it is also possible that youth- and family-level differences across these developmental samples (e.g. youth internalizing levels, attachment styles, parental stress levels) have contributed to the disparate findings across study sites (e.g. Qiu et al., 2015; Rogers et al., 2017; Graham et al., 2018; Scheinost et al., 2016). Future studies may further examine how such factors contribute to amygdala-perigenual mPFC connectivity during development to clarify the prior body of work.

Several caveats should be noted in considering these results. First, large changes in brain size occur during this early developmental period (Huttenlocher and Dabholkar, 1997; Knickmeyer et al., 2008). To address the possible variability in tissue partial volume in different regions, we matched spatial smoothness across subjects (Scheinost et al., 2014). Nonetheless, addressing differential head size in developmental studies remains challenging as a simple covariate for head size (for example) is highly collinear with age. In fMRI generally, and in developmental fMRI in particular, motion has also emerged as a serious potential confound for connectivity studies (Hallquist et al., 2013; Power et al., 2013; Satterthwaite et al., 2012; Scheinost et al., 2014; Power et al., 2015; Ciric et al., 2017), so this study employed very strict motion criteria per current recommendations. However, there is no way presently to index with certainty that no remaining motion-related artifact exists, so this confound may have insidiously impacted the present results (Power et al., 2015; Ciric et al., 2017). The amygdala is also adjacent to ventricles and dense vasculature. This means that, at typical fMRI resolution and sampling rate, there is a risk that the timeseries is confounded by physiological noise (Boubela et al., 2015; Lund et al., 2006; Murphy et al., 2013). We used a data-driven technique to identify and remove structured noise from the fMRI data but, given the concerns about venous signal in particular (Boubela et al., 2015), we also included signal from a population atlas of vascular density (Viviani et al., 2009). However, the vascular maps may have registered with different degrees of success across ages (see Supplemental Fig. 1 for vascular map registration across ages). Our use of the architectonic sub-region maps in infants is also novel, but untested. We checked subregion registration across ages in our sample, and the overlap in findings with studies with youths as young as 4-years through adulthood is consistent with these maps delineating the same sub-regions across development (Alarcón et al., 2015; Kim et al., 2011; Qin et al., 2012; Roy et al., 2013). It should also be noted that the participants in this study were scanned while asleep, as is typical for infant and toddler fMRI studies (e.g. Graham et al., 2015a; Gao et al., 2017). However, sleep state may influence the functional connectivity patterns observed. For example, a recent report suggests sleeping infant fMRI activity most closely resembles that of sleeping adults (Mitra et al., 2017). Prior reports examining whether sleep affects resting-state connectivity patterns are mixed, however, and effects may be region- or network-specific (Fukunaga et al., 2006; Graham et al., 2015b; Horovitz et al., 2008; Horovitz et al., 2009; Larson-Prior et al., 2009; Shen et al., 2016). The impact of sleep state on functional connectivity may be further complicated in pediatric samples like ours by developmental changes in sleep structure across the first few years of life (Roffwarg et al., 1966). Therefore, age-related sleep state changes may have influenced the present findings. Further study of how sleep state may influence connectivity estimates is needed for the infant fMRI field. Additionally the acquisition time for the resting-state scans in the present study was fairly short (∼5 min). Although resting-state connectivity has been shown to stabilize as rapidly as within 5 min (Van Dijk et al., 2010), it is possible that the functional connectivity patterns observed would undergo further changes or stabilization with longer measurements. While the present study was well-powered to detect age-related changes across the sample, there were relatively few participants at any given age, so future studies focusing on more concentrated age-ranges with more participants per age may detect more fine-grained developmental change on shorter scales. Longitudinal studies, that can map out the developmental trajectories of these sub-regions within individuals, are needed to confirm the cross-sectional age-related patterns currently observed. Lastly, the present study had insufficient data to examine how socio-economic status or other early environmental factors may impact the age-related changes observed, but given the amygdala’s sensitivity to early environments in the first few years of life, further research should explore how such factors modulate early amygdala network development (Hackman and Farah, 2009; Tottenham and Gabard-Durnam, 2017).

In summary, these findings illustrate that the amygdala’s LB and SF sub-regions have functional circuits in place by infancy, and progressive shaping of motor and sensory circuit components occurs during this period. Sub-regions can be distinguished from each other by their distinctive connectivity patterns as well as their age-related changes in connectivity. Indeed, these results suggest that the sub-regions may have disparate trajectories of network construction across development, requiring future longitudinal study. The robust amygdala connectivity observed in infancy seems to temporally precede observations of reactivity. These findings present the first information about typical age-related changes in human amygdala functional network development in early childhood and, cumulatively, these results represent an important initial step in understanding the early development of amygdala networks and their dynamics, central to sculpting emotional and social behavior.

## Funding

This work was supported by the National Institutes of Mental Health (RO1 MH087510) and The Bill & Melinda Gates Foundation (0PP1120016) to SD. SD is also supported by the Eunice Kennedy Shriver National Institute of Child Health & Human Development under the National Institutes of Health (NICHD UG3OD023313); LJG-D is supported by the Graduate Research Fellowship from the National Science Foundation (DGE-11-44155). J.O.M. is supported by a Sir Henry Dale Fellowship jointly funded by the Wellcome Trust and the Royal Society (Grant Number 206675/Z/17/Z). This work was supported by the Wellcome EPSRC Centre for Medical Engineering at King's College London (WT 203148/Z/16/Z), the Department of Health through an NIHR Comprehensive Biomedical Research Centre Award (to Guy's and St. Thomas’ National Health Service (NHS) Foundation Trust in partnership with King’s College London and King’s College Hospital NHS Foundation Trust), DCD is supported by a T32 Postdoctoral Training Fellowship awarded by the Eunice Kennedy Shriver National Institute of Child Health & Human Development under the National Institutes of Health (T32HD007489 and U54HD090256) and a National Institute of Mental Health K99 award (K99MH11059).

## Conflict of Interest

None.

## Figures and Tables

**Figure:**
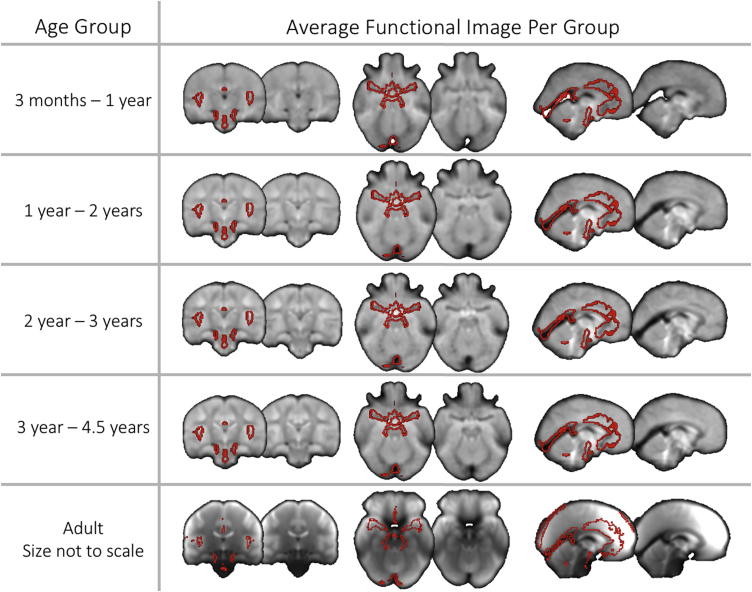


**Figure:**
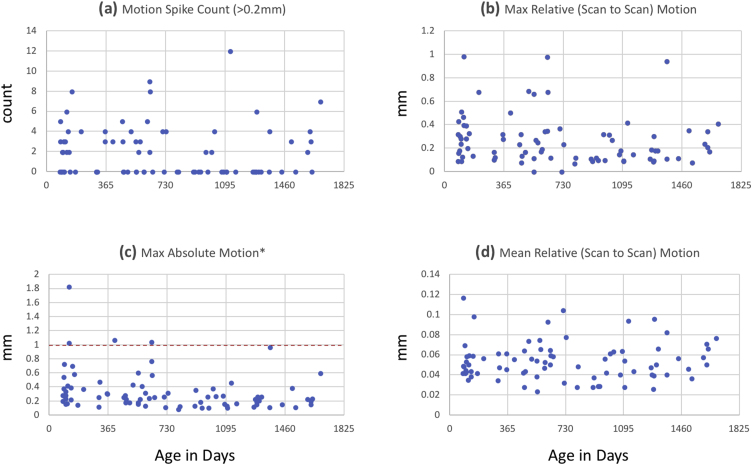


**Figure:**
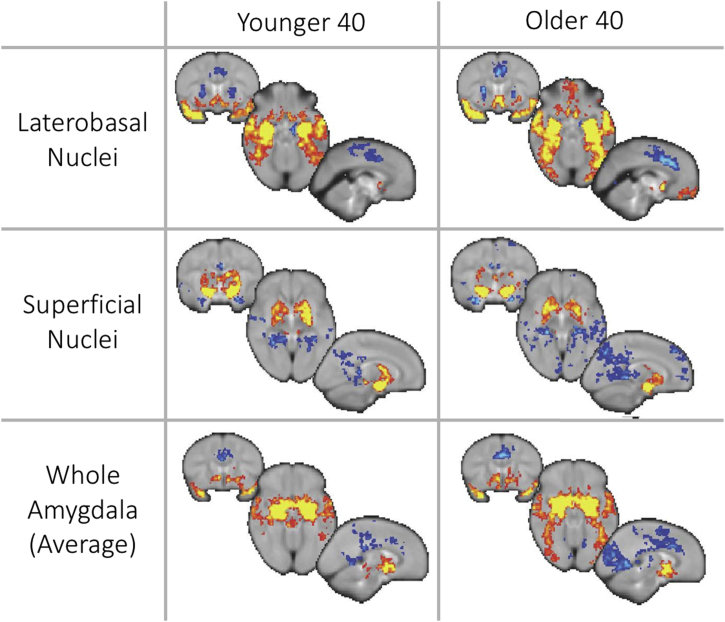


**Figure:**